# Bilayered composite restoration: the effect of layer thickness on fracture behavior

**DOI:** 10.1080/26415275.2020.1770094

**Published:** 2020-06-02

**Authors:** Lippo Lassila, Eija Säilynoja, Roosa Prinssi, Pekka K. Vallittu, Sufyan Garoushi

**Affiliations:** a Department of Biomaterials Science and Turku Clinical Biomaterial Center – TCBC, Institute of Dentistry, University of Turku, Turku, Finland; b Research Development and Production Department, Stick Tech Ltd – Member of GC Group, Turku, Finland; c City of Turku Welfare Division, Oral Health Care, Turku, Finland

**Keywords:** Fracture behavior, bilayered restoration, layer thickness, fiber composite

## Abstract

**Purpose:**

By combining the increased toughness of a resin composite reinforced with discontinuous fibers and the surface wear resistance of a particulate filler composite (PFC), a bilayered composite technique was recently introduced. This study aimed to evaluate the effect of the thickness of the overlaying PFC placed over a fiber-reinforced composite (FRC) core, on the fracture-behavior of direct crown restorations.

**Methods:**

Six groups of posterior crown restorations (*n* = 8/group) were fabricated having a discontinuous FRC-core (everX Flow) and a layer of surface PFC (Essentia U) with various thicknesses (0.5, 1.0, 1.5, 2.0 mm), with the remaining thickness of the bilayered being 6 mm. Control groups were only made of PFC or FRC materials. Restorations were statically loaded until fracture. Failure-modes were visually examined. Data were analyzed using ANOVA (*p* = .05) and regression analysis.

**Results:**

The regression analysis showed that by decreasing the thickness of PFC layer, the load bearing capacity of restorations increased linearly (R^2^=0.7909). ANOVA revealed that crown restorations made only from everX Flow composite had significantly higher load-bearing capacities (3990 ± 331 N) (*p* < .05) among all the groups tested. With regard to the failure-mode analysis, crowns that had a FRC core material of everX Flow revealed delamination of the PFC surface composite from the core. Crowns which were made only of PFC i.e. with no fiber reinforcement, showed a crushing-like fracture pattern.

**Conclusions:**

Restorations combining a thick FRC-core and a thin surface layer of PFC (0.5–1 mm), displayed promising performance related to fracture-behavior and load-bearing capacity.

## 1. Introduction

In the last decades, dental restorative composites have been developed to replace amalgam because of its poor esthetic properties and suggested controversial biocompatibility [1]. Composite restorations have however shown good overall clinical performance in small and medium sized posterior cavities, with annual failure rates being between 1 and 3% [2,3]. The survival of posterior composite restorations strongly correlates with the size of the restorations. Bernardo et al. [4], reported an increase in annual failure rate from 0.95% for single-surface restorations to 9.43% for four or more surface restorations. Large restorations were shown to be more prone to fracture-related failures resulting in decreased longevity [5,6]. Higher susceptibility of large composite restorations to fracture can be attributed to the low fracture toughness of the composite material itself, and patient factors like bruxism [7,8]. Interestingly, Alvanforoush et al. [9], stated that the range of reported overall success rates for long-term clinical studies improved in the period 2006–2016 (minimum 64% to maximum 96.9%) compared with the 1995–2005 (minimum 50% to maximum 83%). However, the reasons for failure have shifted from high rates of secondary caries and wear to increasingly significant roles of restoration fractures, tooth fractures and endodontic treatment [9]. It is clear from the literature that contemporary particulate filled composites (PFCs) still demonstrate limitations because of their insufficient toughness when used in large restorations. Due to failures of this kind, it is still controversial, whether direct restorative PFCs should be used in large high-stress bearing applications such as in core build-ups or posterior crown restorations [1,3].

Several former approaches in the literature have demonstrated the need to find way to support the remaining tooth structure and improve the durability of the final large posterior restorations. One of these attempts has been to use highly tough discontinuous fiber-reinforced composite (FRC) as a core or post-core foundation under surface wear resistance layer of PFC, which can be considered as bilayered composite restorations [10,11].

Although a lot is known about the properties of FRC or PFC itself [12,13], less information is available on the properties of material combination (i.e. bilayered restoration). It can be hypothesized that there are differences in load-bearing capacity and fracture-behavior when the volume ratio of FRC to PFC is changed.

Thus, the aim of this study was to evaluate the influence of thickness ratio of FRC core to the thickness of the overlaying PFC on the load-bearing capacity and fracture-behavior of bilayered direct composite restorations.

## 2. Materials and methods

The materials used in this study are listed in Table 1.

**Table 1.** The commercial composites used.

Material (code)	Manufacturer (Lot No.)	Composition
Essentia, universal shade (PFC)	GC Corp, Tokyo, Japan (1804122)	UDMA, BisEMA, BisGMA, TEGDMA, Bis-MEPP, Prepolymerized silica and barium glass 81 wt%
everX Flow, bulk shade (FRC)	GC Corp, Tokyo, Japan (1810282G)	Bis-EMA, TEGDMA, UDMA, Short glass fiber (200–300 µm and Ø7 μm), Barium glass 70 wt%

Bis-GMA: bisphenol-A-glycidyl dimethacrylate; TEGDMA: triethylene glycol dimethacrylate; UDMA: urethane dimethacrylate; Bis-MEPP: bis (*p*-methacryloxy (ethoxy)1-2 phenyl)-propane; Bis-EMA: ethoxylated bisphenol-A-dimethacrylate; wt%: weight percentage.

### 2.1. Core-crown fabrication

**Figure 1.**
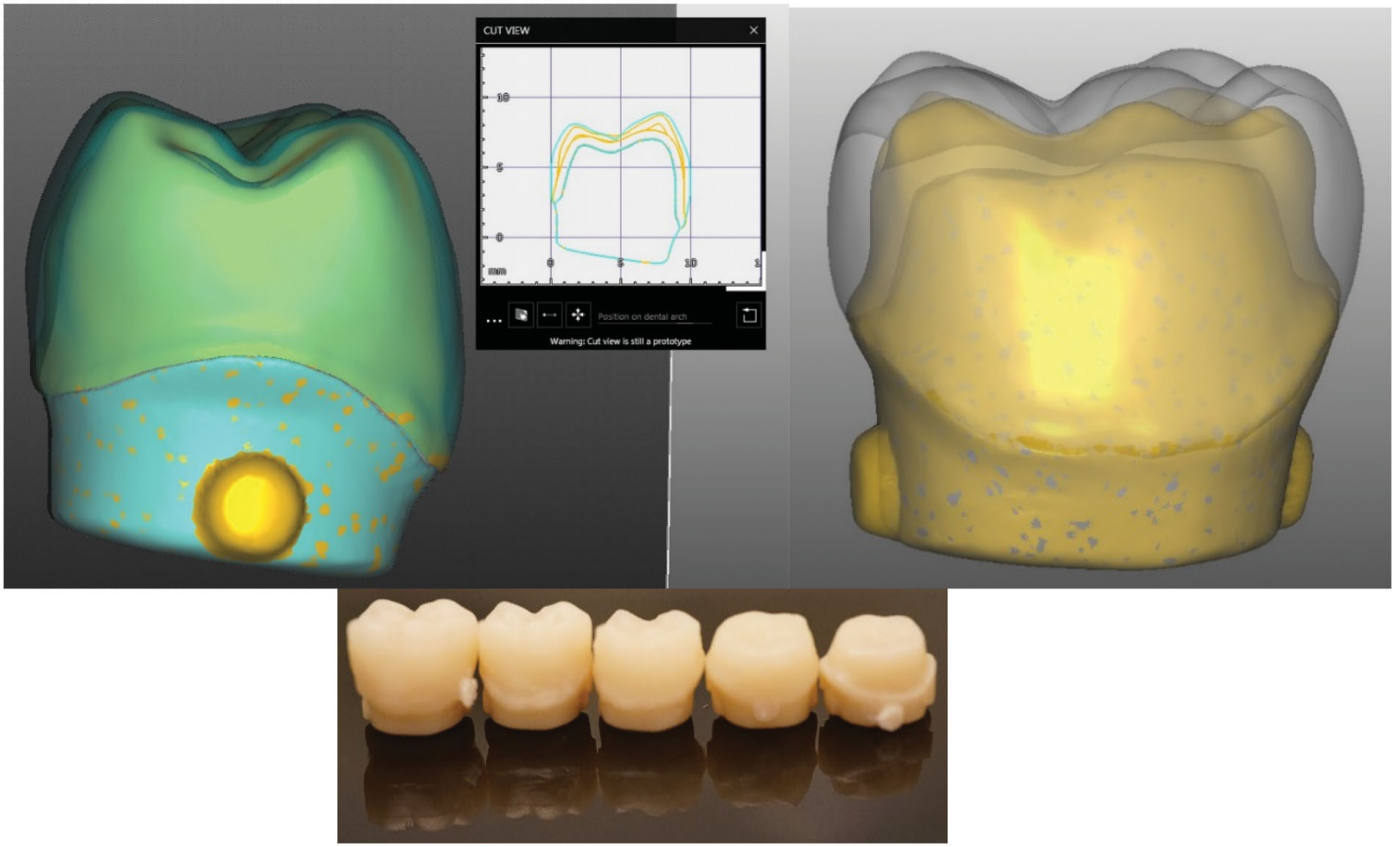
Representation of the process of scanning, designing and milling abutment models with different thicknesses used in this study.

**Figure 2.**
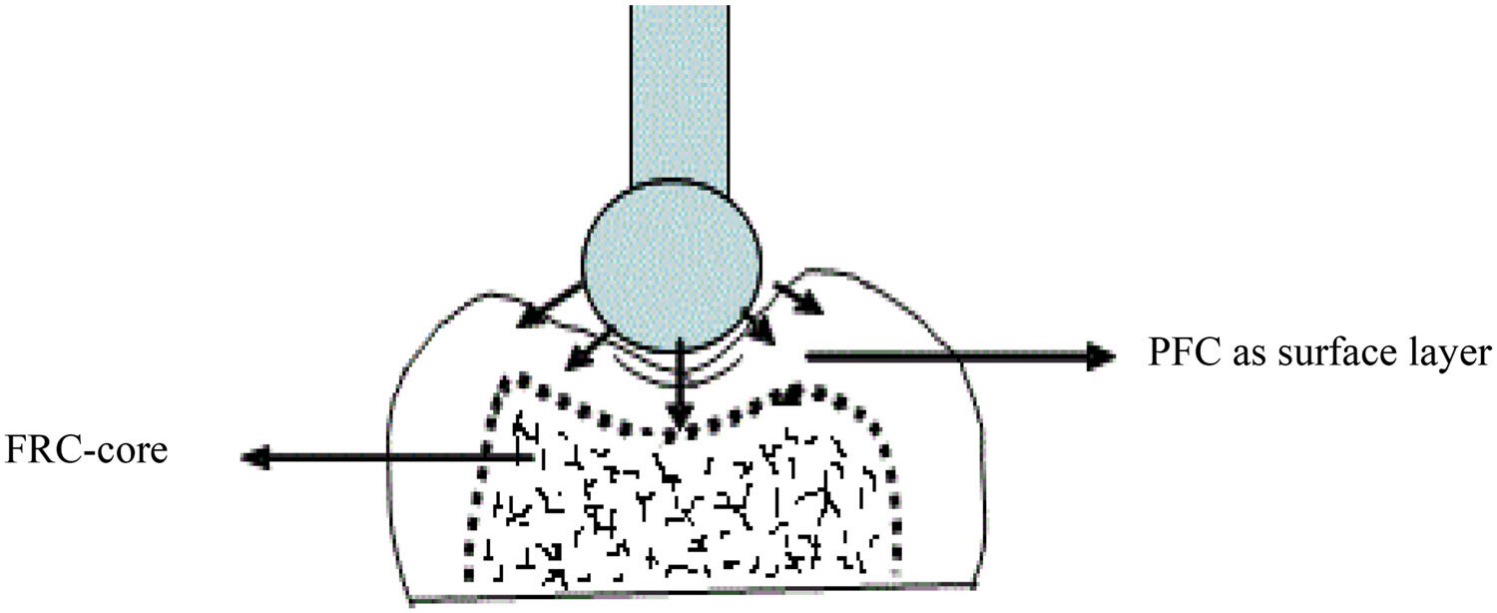
Schematic drawing of a bilayered core-crown restoration and the compression load test setup.

Abutment models with different thicknesses of lower first molar (Frasaco GmbH, Tettnang, Germany) were cut from highly cross linked PMMA blanks (L-Temp MC, DEGOS, Regenstauf, Germany) using a CAD/CAM device (5-TEC, Zirkozahn GmbH, Gais, Italy) (Figure 1). A transparent template index (Memosil 2, Heraeus Kulzer GmbH, Hanau, Germany) of an ideally contoured lower first molar crown (Frasaco) was used to aid standardized core-crown restoration construction. A total of 48 core-crown restorations (bilayered) were constructed having a discontinuous FRC-core and layer of surface PFC with various thicknesses (0.5, 1.0, 1.5, 2.0 mm), remaining the thickness of the bilayered restoration being 6 mm (Figure 2). Control groups were only made of PFC or FRC materials. In order to simulate chair-side fabricated techniques, the PFC pastes were packed into the space created between the transparent index and the abutment models followed by light curing. Then, after removing the abutment from the index, FRC paste directly applied to build-up the core of the restoration.

The core-crown restorations were build-up and light cured incrementally (three-increment) in the same fabricated transparent index. The crown restorations of each group (*n* = 8) were polymerized from all directions using a hand-light curing unit (Elipar TM S10, 3 M ESPE, Seefeld, Germany) for 40 s per increment (wavelength of the light was between 430 and 480 nm and light intensity was 1600 mW/cm^2^). The light source was placed in close contact (1–2 mm) with the composite surface. Prior to testing, all crowns were polished and stored dry for 48 h at 37 °C.

The static compressive fracture test of crown restorations was performed using a universal testing machine (model LRX, Lloyd Instruments Ltd., Fareham, UK) at a speed of 1 mm/min, and data were recorded using PC software (Nexygen Lloyd Instruments Ltd.). The crown was fixed to the flat metal base of the testing device using double sided tape before being statically loaded (spherical Ø 5 mm) (Figure 2). The loading event was registered until restoration fracture (final drop in the load-deflection curve). Failure patterns of each of the loaded restorations were examined visually and categorized into three typical fracture patterns: catastrophic crushing, delamination and cracking [14,15].

### 2.2. Statistical analysis

The data were analyzed using SPSS software version 23(IBM Corp., Somers, NY, USA). The results were primarily analyzed using Levene’s test for equality of variances. When the results of the Levene’s test showed homoscedasticity, values were analyzed using one-way analysis of variance (ANOVA) at the *p < .05* significance level followed by a Tukey HSD *post hoc* test to determine the differences between the groups. A linear regression analysis was used to determine the correlation between the thickness of PFC layer and load-bearing capacity of the test specimens. Correlation was verified by a curve fit test and associated R squared values.

## 3. Results

**Figure 3.**
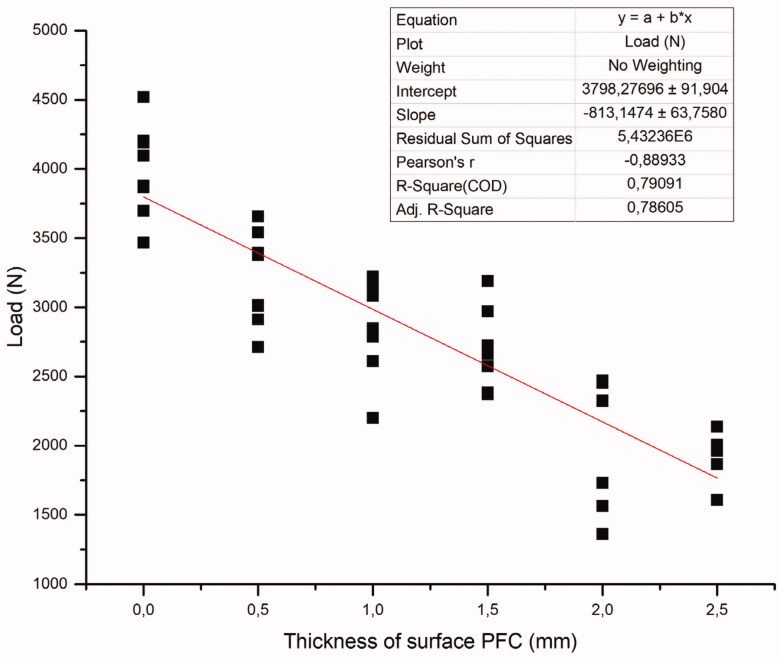
Linear regression (*n* = 48) between different thicknesses of the overlaying PFC and measured load-bearing capacity (N) of tested restorations.

**Figure 4.**
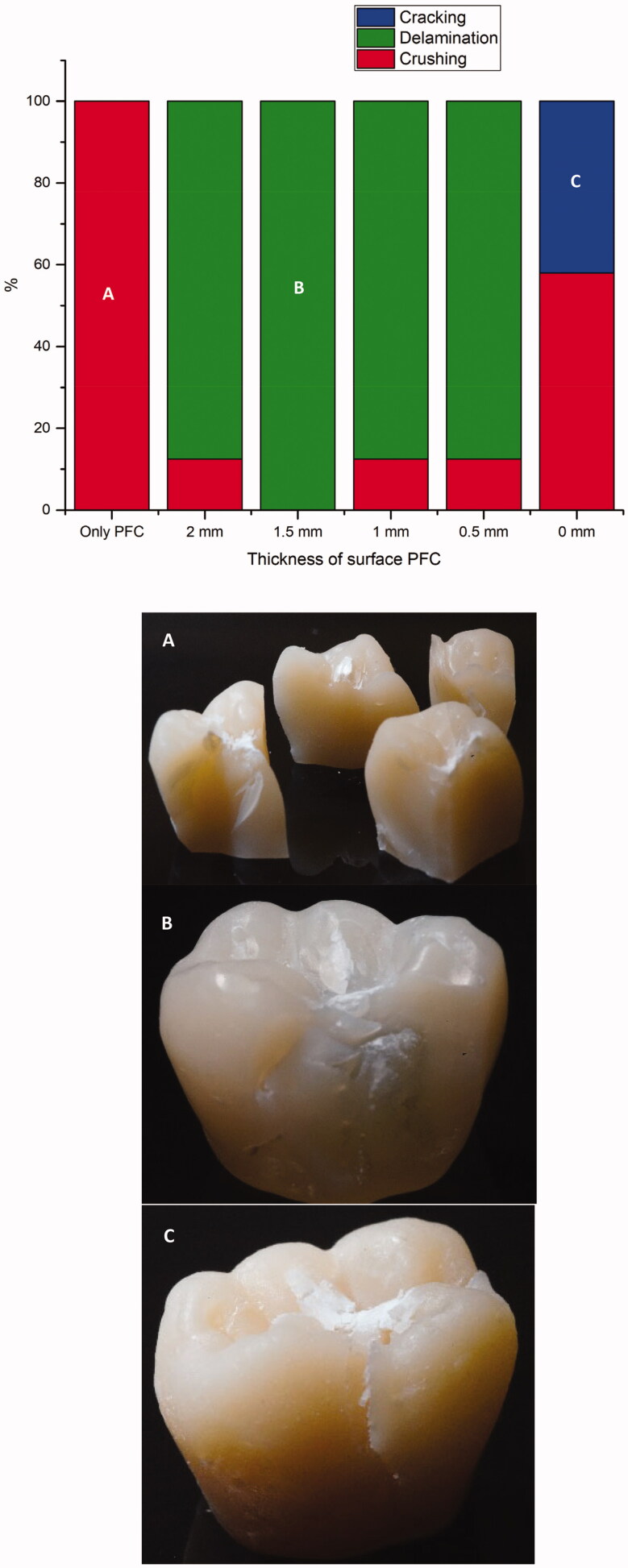
Percentage and photographs of various fracture patterns of the crown specimens. (A) Catastrophic crushing of particulate filler composite; (B) delamination of particulate filler composite from the fiber reinforced core; (C) cracking fracture in plain fiber reinforced composite.

The mean load-bearing capacities of the crown restorations with standard deviations (SD) are given in Table 2. ANOVA revealed that restorations made from plain FRC composite had significantly higher load-bearing capacities (3990 ± 330 N) (*p* < .05) among all the groups tested. Restorations made from FRC-core with a 2 mm thick surface layer of PFC and restorations made from plain PFC had significantly lower load-bearing capacity (*p* < .05) than other groups (20,321,908 N) respectively. The regression analysis relatively showed that by decreasing the thickness of the PFC layer, the load-bearing capacity of restorations increased (R^2^=0.7909) (Figure 3). Curve fit test presented the R squared value (Rs = 0.804; *p* < .005) for the linear regression fit between the two variables. Fracture patterns were analyzed visually, and showed three various types of fracture patterns distributed according to the type of composite core material: catastrophic crushing, delamination and cracking (Figure 4). Crown specimens having only PFC with no fiber reinforcement showed only catastrophic crushing fracture pattern. All of the crown specimens that had a reinforced core material of FRC revealed mostly delaminating of PFC from the FRC-core layer. While the crown specimens that were made from plain FRC, showed catastrophic crushing and cracking fracture patterns (Figure 4).

**Table 2.** Mean fracture load values (N) and standard deviations (SD) of tested restorations with different surface PFC thicknesses.

Thickness of surface PFC	
0 mm	3989.8 (334)^a^
0.5 mm	3202.1 (335)^b^
1 mm	2888.8 (348)^bc^
1.5 mm	2696.5 (278)^c^
2 mm	2032.2 (465)^d^
Only PFC	1907.7 (179)^d^

The same superscript letters represent non-statistically significant differences (*p* > .05) among the groups.

## 4. Discussion

The restorations in this study were designed to evaluate the failure mode and load-bearing capacity of a mandibular first molar restored with a bilayered approach for the fabrication of direct composite crown. In this series an attempt was made by using flowable FRC as core material under surface layer of conventional composite, i.e. bilayered composite restorations.

The data showed substantial improvements in the load-bearing capacity of the restorations when a bulk FRC-core was used compared to that of plain PFC. To some extent, the regression analysis showed that by decreasing the thickness of the PFC layer, the load-bearing capacity of restorations increased linearly (R^2^ = 0.7909) (Figure 3). The function of FRC-core is based on supporting the PFC layer and working as a crack-stopping layer [14]. To receive support from the FRC for the PFC, the structural toughness of the FRC substructure should be higher than that of the PFC surface layer [10,14]. In this, the fiber orientation and cross-linking density of the polymer matrix likely has a significant role. On the other hand, if the function of the FRC-core is based on the mechanism of a crack-stopper, the distance from the surface of the stress initiation point to the fibers is of importance. Therefore, the PFC volume fraction could contribute to the crack propagation and load-bearing capacity. This is in line with previous studies which showed the importance of how thick FRC and PFC layers should be applied [16,17].

Our data showed no statistically significant differences with regard to the load-bearing capacities between crowns made from FRC-core with a 2 mm thick surface layer of PFC, and those made from plain PFCs (Table 2). This is in accordance with previous studies which reported that the incorporation of FRC inside the cavity of posterior destroyed teeth restored with thick PFC composite overlays, was not useful in increasing their load-bearing capacity [18–20]. Since modern composite materials are brittle, they do not lack strength, but they lack toughness [21]. The problem of lack of toughness is especially well seen in extensive direct restorations, as the volume of the PFC material increases [22]. As a result of the above-mentioned disadvantage, direct composite restorations might not be the best solution in a scenario of major loss of tooth structure.

Crown specimens having only particulate filler composite with no fiber reinforcement showed a catastrophic crushing fracture pattern. According to Chai, this seems to be median-radial cracks extending from the ball contact site into the material [23]. It can be clearly seen that the brittleness of the PFC caused the brittle catastrophic fracture. On the other hand, most of the crown specimens that have a reinforced core material of FRC revealed delaminating of PFC from the FRC substructure layer. Hence, the fracture pattern was changed to predominantly favorable or restorable fractures, compared to the plain PFC crown groups. Interestingly, these were similar to natural crown fracture patterns seen in a previous study [15]. Whereas crown specimens that have fabricated from plain FRC showed cracking fracture patterns, which retain the original shape of the crown restoration despite the occurrence of multiple cracks [14]. From a clinical point of view, it is important to consider the consequences of repetitive forces as this pattern of crack would fastly propagate, generating fractures.

Stress applied to teeth and dental restorations is generally low and repetitive rather than being isolated and impactive in nature. However, because of a linear relationship between fatigue and static loading, the compressive static test also gives valuable information concerning the fracture behavior and load-bearing capacity [16,24]. The restorative design used in this study mimicked the scenario of total loss of tooth structure, which considers a limitation with respect to its clinical relevance. Given the mentioned shortcomings, the proposed technique should require future testing with dynamic loading and more clinically relevant design.

## 5. Conclusion

Based on the results of the present in vitro study, one could conclude that optimal thickness of the surface PFC composite over the FRC-core is between 0.5 and 1 mm.
